# Age-Friendly University Assessments: Approach by the UMass Dartmouth Campus

**DOI:** 10.1093/geroni/igaa057.1742

**Published:** 2020-12-16

**Authors:** Andrew Revell, Brian Ayotte, Jennifer Viveiros

**Affiliations:** University of Massachusetts Dartmouth, North Dartmouth, Massachusetts, United States

## Abstract

Representatives from The Ora M. DeJesus Gerontology Center at the University of Massachusetts Dartmouth began participating on the Age-Friendly University (AFU) panel at The Gerontological Society of America conference in November 2018. The recommendations and knowledge provided a foundation to begin informally surveying faculty and gerontology affiliates on age-friendly practices. In the fall of 2019, formal discussions began with our partners at other campuses, and in Spring 2020, the AFU Climate Survey was disseminated by email announcements to all students and employees on our campus, soon followed by the Inventory reporting tool to campus office administrators. Results of these assessments will be presented on the utility of these approaches and suggestions for refinement and broader dissemination.

